# Relación entre consumo moderado de alcohol, polimorfismos genéticos y peso corporal en una muestra poblacional de Puerto Madryn, Argentina

**DOI:** 10.7705/biomedica.7270

**Published:** 2024-11-06

**Authors:** Luis Orlando Pérez, Anahí Ruderman, Mariana Useglio, Virginia Ramallo, Carolina Paschetta, Soledad de Azevedo, Pablo Navarro, Leonardo Morales, Magda Alexandra Trujillo-Jiménez, Bruno Pazos, Tamara Teodoroff, Rolando González-José

**Affiliations:** 1 Instituto Patagónico de Ciencias Sociales y Humanas, CCT CENPAT-CONICET, Puerto Madryn, Argentina Instituto Patagónico de Ciencias Sociales y Humanas Puerto Madryn Argentina; 2 Programa de Referencia y Biobanco Genómico de la Población Argentina, Secretaría de Planeamiento y Políticas, Ministerio de Ciencia, Tecnología e Innovación, Ciudad Autónoma de Buenos Aires, Argentina Secretaría de Planeamiento y Políticas Buenos Aires Argentina; 3 Laboratorio de Ciencias de las Imágenes, Departamento de Ingeniería Eléctrica y Computadoras, Universidad Nacional del Sur, Bahía Blanca, Buenos Aires, Argentina Universidad Nacional del Sur Departamento de Ingeniería Eléctrica y Computadoras Universidad Nacional del Sur Buenos Aires Argentina; 4 Departamento de Informática, Facultad de Ingeniería, Universidad Nacional de la Patagonia San Juan Bosco, Trelew, Argentina Universidad Nacional de la Patagonia San Juan Bosco Departamento de Informática Facultad de Ingeniería Universidad Nacional de la Patagonia San Juan Bosco Trelew Argentina

**Keywords:** consumo de bebidas alcohólicas, índice de masa corporal, polimorfismo genético, metabolismo, factores de riesgo, obesidad, Alcohol drinking, body mass index, polymorphisms, genetic, metabolism, risk factors, obesity

## Abstract

**Introducción.:**

La relación entre la obesidad y el consumo de alcohol es un tema de gran interés para la salud pública. Las bebidas alcohólicas aportan calorías adicionales a la dieta, lo que podría ser un factor relevante en el riesgo de sobrepeso. Sin embargo, su asociación con la ganancia de peso es controversial y está influenciada por múltiples factores.

**Objetivo.:**

Analizar la relación entre la ingestión moderada de alcohol y el índice de masa corporal, y las variables que pueden influir en dicha relación.

**Materiales y métodos.:**

La muestra estuvo constituida por 155 personas de Puerto Madryn (Argentina). Cada participante contestó un cuestionario sobre salud, estilo de vida, factores demográficos y socioeconómicos. Se tomaron medidas antropométricas y se tipificaron los polimorfismos de 18 genes relacionados con el metabolismo del alcohol.

**Resultados.:**

Se encontró que el consumo moderado de alcohol está asociado con un índice de masa corporal más bajo, particularmente en el sexo femenino. Un aumento de 14 gramos de alcohol estuvo asociado con un riesgo de 0,68 para la obesidad y 0,71 para el sobrepeso. La variante T del marcador *rs4646543 (ALDH1A1),* un gen involucrado en el metabolismo del alcohol y en la adipogénesis, se asoció con una mayor frecuencia de consumo de bebidas alcohólicas.

**Conclusión.:**

Los hallazgos del presente trabajo sugieren que el consumo moderado de alcohol no contribuye significativamente al peso corporal en la muestra estudiada. Además, la asociación con ciertas variantes genéticas, como las del gen *ALDH1A1* , podría explicar biológicamente la relación inversa observada entre el peso y el consumo de alcohol.

## Abstract (en)

**Introduction.:**

The relationship between obesity and alcohol consumption is a topic of significant interest to public health. Alcoholic beverages contribute additional calories to the diet, which could be a relevant factor to the overweight risk. However, its association with weight gain is controversial and influenced by multiple factors.

**Objective.:**

To analyze the relationship between moderate alcohol intake and body mass index, considering the variables that may influence this relationship.

**Materials and methods.:**

The sample consisted of 155 individuals from Puerto Madryn (Argentina). Each participant completed a questionnaire about health, lifestyle, demographic, and socioeconomic factors. Anthropometric measurements were taken, and polymorphisms of 18 genes related to alcohol metabolism were genotyped.

**Results.:**

We found that moderate alcohol consumption is associated with a lower body mass index, particularly in females. An increase of 14 grams of alcohol was associated with a risk of 0.68 for obesity and 0.71 for overweight. The T variant of the marker *rs4646543 (ALDH1A1* ), a gene involved in alcohol metabolism and adipogenesis, was associated with a higher frequency of alcohol consumption.

**Conclusion.:**

The findings of this study suggest that moderate alcohol consumption does not significantly contribute to body weight in the sample studied. Furthermore, the association with genetic variants, such as those of the *ALDH1A1* gene, may provide a biological explanation for the inverse relationship observed between weight and alcohol consumption.

El aumento del consumo de alcohol es una tendencia preocupante que afecta la salud y el bienestar de la población. Según datos de la Organización Mundial de la Salud, Argentina es el segundo país en consumo *per cápita* en Latinoamérica, solo superado por Uruguay ^1^^,^^2^. Se estima un consumo promedio por persona -mayor de 15 años- de 9,88 litros de alcohol puro al año, mientras que el promedio regional es de 8 litros. Según la Cuarta Encuesta Nacional de Factores de Riesgo (2019), los hombres consumen más que las mujeres, tanto en cantidad como en frecuencia ^3^. El vino, que solía ser la bebida alcohólica más popular en Argentina, ha dado su lugar a la cerveza, cuya popularidad ha crecido rápidamente en los últimos años, especialmente en el segmento juvenil ^4^.

El sobrepeso y la obesidad son condiciones complejas que se han asociado con el consumo de alcohol. Este vínculo se produce por la inhibición de la oxidación de grasas, la ingestión adicional de calorías, la estimulación del apetito, y el aumento de consumo de alimentos salados y grasos ^5^^-^^7^.

No obstante, desde el punto de vista epidemiológico, la evidencia es controversial, pues también hay indicios de un efecto protector del alcohol sobre la ganancia de peso. El consumo intenso o serio de alcohol puede acarrear un mayor riesgo de sobrepeso que el regular, mientras que el consumo leve a moderado -especialmente de vino- parece tener un efecto variable sobre el peso, dependiendo de la población ^8^^,^^9^. Hay consenso en que los episodios serios pero breves, también llamados "atracones de alcohol", están asociados con el aumento de peso o de la circunferencia de la cintura ^10^.

A nivel fisiológico, existe una considerable variación individual en la reacción ante las bebidas alcohólicas. El alcohol es oxidado en el hígado a acetaldehído por la enzima alcohol deshidrogenasa (ADH) y, posteriormente, a acetato por la aldehído deshidrogenasa (ALDH) ^11^. El acetaldehído es un intermediario tóxico y reactivo, y su acumulación provoca síntomas adversos, incluyendo el síndrome de rubor, taquicardia y náuseas, entre otros ^12^. Se ha observado que dichas enzimas están involucradas con la sensibilidad y dependencia del alcohol, en particular, ciertas variantes del gen *ADH1B* en poblaciones europeas y asiáticas, o bien variantes del gen *ALDH2* que metabolizan el acetaldehído a menor velocidad y generan efectos desagradables, como el eritema transitorio ^13^.

Debido a su mecanismo de acción, las enzimas desintoxicantes solapan sus funciones con la regulación de otras vías, relacionadas con el apetito y el peso corporal. Por ejemplo, la enzima ALDH1A1 sintetiza ácidos retinoicos que son reguladores importantes de la adipogénesis, evento que está asociado con la expresión de la adiponectina y la inhibición de la formación de adipocitos ^14^.

El objetivo del presente estudio fue analizar en un conjunto de personas que viven en Puerto Madryn (Argentina) cuál es la relación entre la ingestión de alcohol autorreportada y el índice de masa corporal, considerando variables socioeconómicas y el efecto de los polimorfismos de los genes en el metabolismo de alcohol.

## Materiales y métodos

### Población

Se hizo una convocatoria abierta durante el 2018 en Puerto Madryn para participar en un estudio sobre enfermedades complejas. El proyecto fue publicitado en el portal web oficial, redes sociales y medios de comunicación locales. La muestra seleccionada estuvo compuesta por 155 personas entre los 18 y los 70 años. El diseño del estudio fue transversal y el tipo de muestreo fue deliberado. Se estableció como criterio de inclusión la mayoría legal de edad y el lugar de nacimiento, que debía ser Argentina o alguno de sus países limítrofes.

A cada participante se le hizo una entrevista personalizada, que abordó variables de salud, actividad física, educación, factores socioeconómicos, dieta, tabaquismo y consumo de bebidas, entre otros. Para la construcción de la variable socioeconómica, se analizaron los componentes principales con las preguntas sobre ingresos, características de vivienda y habitabilidad, servicios, movilidad y conectividad. La variable se dividió en cuartiles para su análisis.

Las mediciones antropométricas realizadas incluyeron la circunferencia de la cintura (estimada como el punto medio entre el margen costal inferior y el borde superior de la cresta ilíaca sobre la línea axilar media) y la circunferencia de la cadera (el punto más ancho de los glúteos). Para la toma de las medidas, se utilizó una cinta métrica ergonómica Seca 201 (Seca GmBH & Co Kg, Hamburgo, Alemania) con divisiones de 1 mm y un rango de 0 a 205 cm. La altura total se midió con la cinta métrica mecánica Seca 206 (Seca GmBH & Co Kg, Hamburgo, Alemania), con una longitud de graduación de 1 mm y un rango de 0 a 220 cm. El peso total y la composición corporal (masa muscular, masa magra, grasa corporal y porcentajes) se estimaron con una báscula de bioimpedancia (Tanita BC 1100F).

Para controlar posibles errores intraobservador, todas las mediciones se obtuvieron por duplicado con un criterio de tolerancia de no más de 0,5 cm. La medida promedio se utilizó en los análisis posteriores. Se determinó el índice de masa corporal como la relación entre el peso y el cuadrado de la talla (kg/m^2^). Además de las mediciones antropométricas (circunferencias de la cintura y cadera, talla y peso), se tomaron muestras de sangre por punción venosa para tipificar los polimorfismos de ADN.

### Medidas de alcohol

Se registró la frecuencia de consumo de alcohol (días por semana), en número de copas según la bebida y en unidades estándar de alcohol, determinadas como 14 g de alcohol puro de acuerdo con los estándares del Ministerio de Salud de la Nación ^15^. La medida de la copa de vino se estableció en 150 ml, la de cerveza en 330 ml y las de bebidas fuertes en 44 ml. Se consideró como consumo moderado hasta dos unidades estándar de bebida por día (28 g de alcohol/día).

### Extracción de ADN y genotipificación

Se extrajo ADN a partir de 8 ml de sangre mediante una técnica de extracción con sales de gran concentración.

Brevemente, se lisaron los glóbulos rojos con solución RBC (1 mM de bicarbonato de amonio y 115 mM de cloruro de amonio) y, posteriormente, los glóbulos blancos con solución WCL (100 mM de Tris-HCL, 40 mM de EDTA, 50 mM de NaCl, 0,2% de SDS y 0,05% de azida sódica). El sobrenadante fue precipitado con 2,5 M de NaCl, purificado con isopropanol (2:1) y rehidratado con agua destilada.

Noventa y seis muestras elegidas al azar fueron tipificadas mediante el set de microarreglos *Precision Medicine Array™* (Affymetrix, USA) para realizar un estudio piloto con los siguientes marcadores de genes del metabolismo del alcohol y uno de obesidad: *rs4646487 (CYP4B1); rs17417191, rs4646892 (ALDH9A1); rs1061187 (ADH5); rs1126673(ADH4); rs5860571 (ADH6); rs1042026 (ADH1B); rs283413, rs3114047 (ADH1C); rs4147553, rs284785 (ADH7); rs45621431 (CYP3A43); rs6952940 (TBXAS1); rs4646543, rs8187876 (ALDH1A1); rs2249694 (CYP2E1); rs11032699, rs2284368 (CAT); rs2238151 (ALDH2); rs2472304 (CYP1A2); rs59755039 (ALDH3A2); rs2108622 (CYP4F2); rs11075997y rs9939609 (FTO).*

A partir de los resultados, se seleccionaron cuatro marcadores de interés y se tipificaron todas las muestras con la técnica PCR alelo-específica. Las secuencias de los oligonucleótidos, el tamaño de los fragmentos y la información adicional, se encuentran disponibles en el cuadro 1.

**Cuadro 1:** Oligonucleótidos utilizados para amplificación de ADN

Nombre	Secuencia	Tamaño (pb)	Gen, variante y posición
For.876.A	CTG TTT CCT ACA AGT ATG CCT CAC A	167	*ALDH1A1*
For.876.G	CTG TTT CCT ACA AGT ATG CCT CAC G		*rs8187876*
Rev. 876	GCA AAC AAA GTT ACC GAA TCC		Cromosoma 9
			Posición 72950038
For.543.T	GTG GTC AAA TGC TTG GAA TAA ACC TAA T	350	*ALDH1A1*
For.543.C	GTG GTC AAA TGC TTG GAA TAA ACC TAA C		*rs4646543*
Rev. 543	GCT GGA TGG GTA GCT AGA GC		Cromosoma 9
			Posición 72930540
For.997.C	GTA TTA GGG AAG GGA ATT GAC C	341	*FTO*
For.997.T	GTA TTA GGG AAG GGA ATT GAC T		*rs11075997*
Rev2.997	TGG ACA CCA ACC CCT GGC T		Cromosoma 16
			Posición 53825000
For.609.T	CCT TGC GAC TGC TGT GAA TTT T	250	*FTO*
For.609.A	CCT TGC GAC TGC TGT GAA TTT T		*rs9939609*
Rev2.609	CAC TGC GCC CAG CCC AAG GAT		Cromosoma 9
			Posición 53786615

pb: pares de bases

Para el diseño de los *primers* se utilizó el *software* FastPCR. La mezcla de reacción consistió en 1,5 a 2 mM de MgCl_2_, 1 μM de cada oligonucleótido, 200 μM de dNTPs, 1X de solución tampón comercial y 1 unidad de Taq DNA polimerasa (Inbio^TM^, Argentina). El ciclo fue el mismo en todos los casos y consistió en desnaturalización inicial a 95 °C por dos minutos, seguida de 33 ciclos de 92 °C por 30 s, 60 °C por 20 s y 72 °C por 30 s, más una amplificación final a 72 °C por cinco minutos.

### Análisis estadístico

La descripción de la muestra se presentó como promedios para las variables cuantitativas y, como frecuencias y tablas cruzadas, para las variables cualitativas. Para determinar si existían diferencias estadísticamente significativas entre los grupos de las variables, se utilizó la prueba de Kruskal-Wallis y, para la distribución de frecuencias, la prueba de ji al cuadrado (X^2^).

Se utilizaron tres modelos de regresión lineal múltiple. En cada uno se empleó una variable dependiente diferente: la frecuencia semanal, la cantidad de alcohol o el índice de masa corporal. Las variables independientes fueron: sexo, edad, nivel socioeconómico, educación formal (medida como el último grado completado por cada persona en cualquiera de los cuatro niveles educativos: secundario, terciario, universitario y posuniversitario), consumo de tabaco y actividad física.

Para evaluar el riesgo de sobrepeso y obesidad por el consumo de alcohol, se emplearon regresiones logísticas. En estos modelos, el índice de masa corporal (IMC) se consideró como variable dependiente, codificada según los criterios de 'normal Vs. sobrepeso' en uno y 'normal Vs. obesidad' en otro. El consumo de alcohol y demás covariables se incluyeron como independientes.

Para seleccionar los marcadores de interés, se utilizó el modelo lineal generalizado incluido dentro del *software* Plink ^16^. Los demás análisis y gráficos se llevaron a cabo con el paquete estadístico R ^17^.

### Aspectos éticos

El estudio fue aprobado por el Comité de Bioética de la Región Programática Norte de la provincia de Chubut (Resolución 19/17) y se obtuvo el consentimiento libre e informado por escrito de cada participante. El consentimiento incluyó información sobre la finalidad del estudio, el proceso de anonimidad de los datos, la protección de la identidad y los derechos de la persona donante.

## Resultados

### Características generales de la muestra

Las características principales de la muestra están descritas en el cuadro 2. Las mujeres comprendieron el 67 % (105/155), mientras que el 33 % restante fueron hombres. El promedio de edad fue de 39 años (rango: 21 - 69), sin diferencias significativas entre mujeres (40,8 años) y hombres (37,9 años) (t [137] = 1,71; p = 0,1).

El grado de educación formal se distribuyó de manera homogénea en los cuatro niveles. En cuanto a la actividad física, casi la mitad de la muestra (45,81 %; 71/155) declaró practicar de dos a tres días a la semana algún deporte, caminar o desplazarse en bicicleta. El 10,32 % manifestó hacerlo diariamente. En promedio, los hombres fueron menos activos que las mujeres. La mitad de las personas entrevistadas fumó o había dejado de fumar en los últimos diez años.

El promedio del índice de masa corporal para toda la muestra fue de 27,9 (rango: 17,7 - 47,87; DE = 6,1), sin diferencias significativas entre mujeres (28,0) y hombres (27,6) (t [139] = 0,47; p = 0,63).

### Consumo de alcohol

Al considerar el total de la muestra, el consumo promedio fue de 4,43 unidades de alcohol por semana. Los hombres consumieron más, en cantidad y en frecuencia, registrando 6,69 unidades de alcohol por semana, mientras que las mujeres reportaron 3,35 unidades de alcohol. Si se consideran solamente a los bebedores, los valores cambian a 7,27 y 4,14 unidades de alcohol para hombres y mujeres, respectivamente (cuadro 2). Estos datos indican un radio de 1,75 a casi 2 entre sexos. El análisis de Kruskal-Wallis no evidenció diferencias significativas de consumo entre clases de edad, tanto en hombres (H [3] = 2,6; p = 0,45) como en mujeres (H[3] = 3,8; p = 0,28).

**Cuadro 2:** Características de la muestra

		Total		Hombres		Mujeres	
		media ± DE	n (%)	media ± DE	n (%)	media ± DE	n (%)
Muestra completa			155 (100)		50 (32,3)		105 (67,7)
IMC (kg/m^2^)		27,9 ± 6,13		27,62 ± 4,04		28,05 ± 6,95	
Talla (cm)		163,5 ± 12,67		175,08 ± 6,2		158,05 ± 11,2	
Cintura (cm)		94,04 ± 17,07		96,22 ± 10,86		92,98 ± 19,34	
Edad (años)		39 ± 11,47		37,88 ± 8,33		40,79 ± 12,62	
Episodios por semana (frecuencia)							
	Toda la muestra	1,69 ± 1,52		2,09 ± 1,72		1,50 ± 1,39	
	Solo bebedores	2 ± 1,46		2,27 ± 1,67		1,85 ± 1,32	
Unidades de alcohol por semana							
	Toda la muestra	4,43 ± 4,66		6,69 ± 5,85		3,35 ± 3,5	
	Solo bebedores*	5,24 ± 4,63		7,27 ± 5,74		4,14 ± 3,46	
Grupos de edad							
	20 - 30		30 (19,35)		6 (12,00)		24 (22,86)
	> 30 - 40		60 (38,72)		27 (54,00)		33 (31,43)
	> 40 - 50		34 (21,93)		13 (26,00)		21 (20,00)
	> 50		31 (20,00)		4 (8,00)		27 (25,71)
Nivel de educación							
	1		40 (25,80)		10 (20,00)		30 (28,57)
	2		42 (27,10)		12 (24,00)		30 (28,57)
	3		35 (22,58)		13 (26,00)		22 (20,95)
	4		38 (24,52)		15 (30,00)		23 (21,91)
Actividad física							
	No		38 (24,52)		12 (24,00)		26 (24,76)
	Ocasional^a^		30 (19,35)		12 (24,00)		18 (17,14)
	Regular^b^		71 (45,81)		22 (44,00)		19 (46,67)
	Todos los días		16 (10,32)		4 (8,00)		12 (11,43)
Consumo de tabaco							
	No		73 (47,09)		25 (50,00)		48 (45,71)
	En el pasado		50 (32,26)		16 (32,00)		34 (32,38)
	Sí		32 (20,65)		9 (18,00)		23 (21,91)
Nivel socioeconómico^c^							
	1		39 (25,16)		17 (34,00)		22 (20,95)
	2		39 (25,16)		15 (30,00)		24 (22,86)
	3		39 (25,16)		8 (16,00)		31 (29,52)
	4		38 (24,52)		10 (20,00)		28 (26,67)

DE: desviación estándar; IMC: índice de masa corporal

Nota. Nivel de educación: 1) estudios secundarios, 2) terciarios, 3) universitarios y 4) posuniversitarios Actividad física: ^a^ ocasional: 1 a 2 veces por semana, ^b^ regular: 3 veces por semana.

^c^ El nivel socioeconómico fue ordenado del cuartil menor a mayor. *Solo bebedores: se refiere a la submuestra integrada por las personas que expresaron haber tomado alcohol.

**Figura 1:**
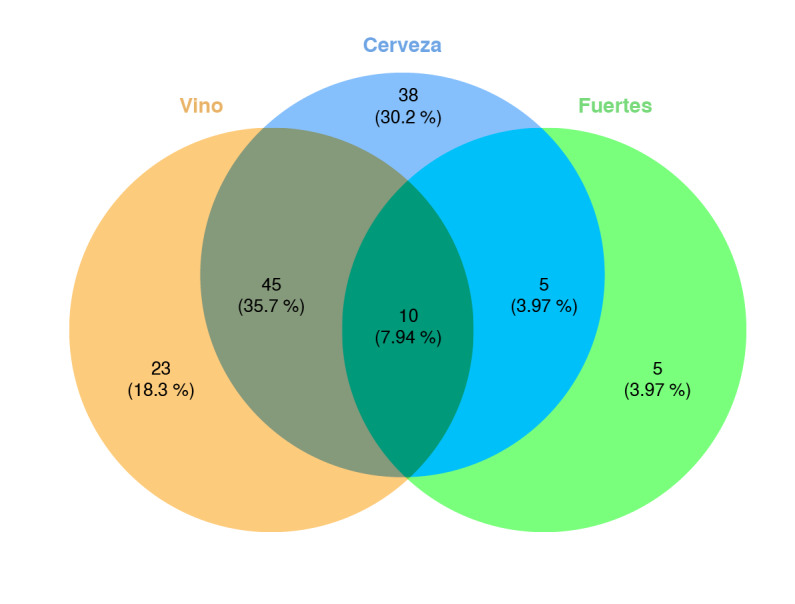
Diagrama de Venn que representa el solapamiento del consumo de bebidas alcohólicas

Como una característica general, hombres y mujeres tomaron más cerveza que vino y las bebidas fuertes estuvieron en último lugar. Además, muchos de los participantes de este estudio toman más de un tipo de bebida alcohólica: la combinación de cerveza y vino se informó en el 35,7 % de las entrevistas (figura 1).

En cuanto a la frecuencia de consumo de alcohol, los dos sexos estuvieron más representados en el rango de menor consumo, de uno hasta cuatro veces por semana. Sin embargo, los hombres consumieron mayor cantidad si se consideran más de cuatro unidades de alcohol por semana (cuadro 3).

**Cuadro 3:** Frecuencia de copas y cantidad de alcohol por semana

		Total	Hombres	Mujeres	X^2^
		n (%)	n (%)	n (%)	(p)
Número de episodios por semana (frecuencia)					
	0	24 (15,48)	4 (8,00)	20 (19,05)	1,56 (0,45)
	1	66 (42,58)	21 (42,00)	45 (42,86)	
	2	36 (23,23)	12 (24,00)	24 (22,86)	
	> 3	29 (18,71)	13 (26,00)	16 (15,23)	
Unidades de alcohol por semana					
	0	24 (15,48)	4 (8,00)	20 (19,05)	14,8 (0,002)*
	> 0 - 2	28 (18,06)	5 (10,00)	23 (21,90)	
	> 2 - 4	44 (28,39)	10 (20,00)	34 (32,38)	
	> 4 - 6	21 (13,55)	10 (20,00)	11 (10,48)	
	> 6	38 (24,52)	21 (42,00)	17 (16,19)	
Copas de cerveza (330 ml) por semana					
	0	53 (34,19)	11 (22,00)	42 (40,00)	16,62 (8,4x10^-4^)*
	> 0 - 1	17 (10,97)	2 (4,00)	15 (14,28)	
	> 2 - 4	39 (25,16)	13 (26,00)	26 (24,76)	
	> 4 - 6	22 (14,2)	7 (14,00)	15 (14,29)	
	> 6	24 (15,48)	17 (34,00)	7 (6,67)	
Copas de vino (200 ml) por semana					
	0	75 (48,39)	20 (40,00)	55 (52,38)	8,5 (0,03)*
	1	21 (13,55)	4 (8,00)	17 (16,19)	
	2	29 (18,71)	9 (18,00)	20 (19,05)	
	3 - 4	13 (8,38)	8 (16,00)	5 (4,76)	
	> 5	17 (10,97)	9 (18,00)	8 (7,62)	
Copas de bebidas fuertes (45 ml) por semana					
	0	135 (87,10)	44 (88,00)	91 (86,67)	3,05 (0,21)
	1	11 (7,10)	1 (2,00)	10 (9,52)	
	2	4 (2,58)	2 (4,00)	2 (1,91)	
	3 - 4	2 (1,29)	1 (2,00)	1 (0,95)	
	> 4	3 (1,93)	2 (4,00)	1 (0,95)	

* p < 0,05

### Estudios de asociación

## Relación entre índice de masa corporal y consumo de alcohol

**Figura 2:**
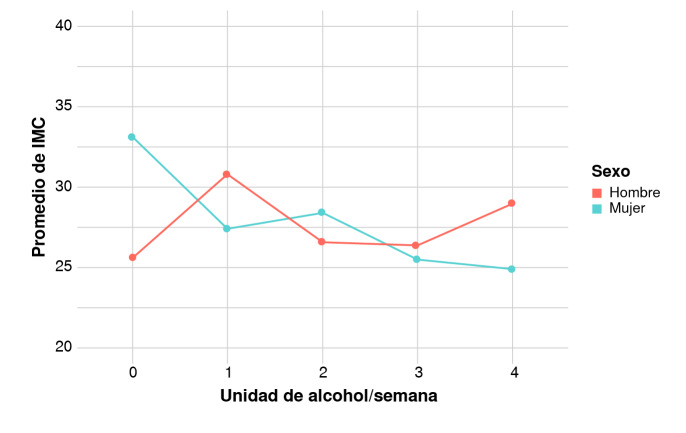
A) Promedio del índice de masa corporal según las unidades de alcohol (14 g) consumidas por semana. B) Promedio del índice de masa corporal según la frecuencia de consumo de alcohol

Los resultados de los modelos de regresión lineal se presentan en el cuadro 4. Se encontró que un incremento en la frecuencia del consumo y en la cantidad de alcohol, está asociado con un menor índice de masa corporal en la población estudiada. Al dividir la muestra por sexo, esta relación se mantuvo solamente en las mujeres. La figura 2 muestra cómo el promedio de IMC va disminuyendo según el incremento del consumo de alcohol, utilizando valores combinados de vino, cerveza y bebidas fuertes. Respecto a las variables socioeconómicas, la frecuencia de consumo de alcohol estuvo asociada con un menor nivel socioeconómico y a un mayor nivel educativo, mientras que la cantidad de alcohol no estuvo asociada con las covariables.

**Cuadro 4:** Regresiones lineales múltiples de la frecuencia y la cantidad de alcohol por sexo

	Frecuencia de consumo (días por semana)			Cantidad de consumo (unidades de alcohol por semana)		
Variables	Conjunto	Mujeres	Hombres	Conjunto	Mujeres	Hombres
	ß (p)	ß (p)	ß (p)	ß (p)	ß (p)	ß (p)
Edad	-0,01 (0,61)	0,006 (0,58)	-0,008 (0,8)	-0,01 (0,78)	0,01 (0,68)	-0,09 (0,34)
Educación	0,29 (0,006)*	0,28 (0,03)*	0,2 (0,25)	0,39 (0,24)	0,26 (0,49)	0,68 (0,31)
IMC	-0,05 (0,03)*	-0,06 (0,01)*	-0,02 (0,6)	-0,09 (0,20)	-0,16 (0,03)*	0,20 (0,32)
Nivel socioeconómico	-0,14 (0,02)*	-0,11 (0,04)*	-0,05 (0,7)	-0,28 (0,15)	-0,34 (0,13)	-0,24 (0,61)
Actividad física	-0,05 (0,69)	0,01 (0,9)	-0,2 (0,39)	-0,3 (0,46)	-0,30 (0,51)	-0,38 (0,64)

IMC: índice de masa corporal * p < 0,05

A partir de los modelos de regresión logística, se pudo estimar que el incremento en la frecuencia del alcohol estuvo asociado con un *odds ratio* de 0,68 (IC_95%_: 0,49 - 0,95; p = 0,02) para la obesidad, y de 0,71 (IC_95%_: 0,56 -0,91; p = 0,001) para el sobrepeso. Por otra parte, el incremento de una unidad de alcohol a la semana estuvo asociado con un *odds ratio* de 0,94 (IC_95%_: 0,87 - 1,01; p = 0,11) para sobrepeso y 0,92 (IC_95%_: 0,84 - 1,01; p = 0,11) para obesidad, aunque no de manera estadísticamente significativa (cuadro 5).

**Cuadro 5:** Regresiones logísticas del efecto de la frecuencia y la cantidad de alcohol semanal sobre la obesidad y el sobrepeso, ajustado por edad y sexo

	Frecuencia			Cantidad		
	ß	OR (IC^95%^)	z (p)	ß	OR (IC^95%^)	z (p)
Obesidad						
Educación	-0,4	0,66 (0,46-0,94)	-2,29 (0,02)*	-0,45	0,63 (0,44-0,89)	-2,57 (0,01)*
SES	0,13	1,14 (0,92-1,41)	1,21 (0,22)	0,12	1,13 (0,91-1,40)	1,14 (0,25)
Actividad física	-0,46	0,66 (0,43-1,01)	-1,88 (0,05)^sl^	-0,41	0,66 (0,43-1,01)	-1,88 (0,05)^sl^
Alcohol	-0,5	0,68 (0,49-0,95)	-2,29 (0,02)*	-0,06	0,92 (0,84-1,01)	-1,36 (0,11)
Sobrepeso						
Educación	-0,12	0,88 (0,63-1,22)	-0,74 (0,45)	-0,21	0,81 (0,59-1,01)	-1,36 (0,17)
SES	0,2	1,22 (1,01-1,47)	2,08 (0,03)*	0,17	1,18 (0,98-1,41)	1,83 (0,06)
Actividad física	-0,2	0,81 (0,54-1,21)	-0,99 (0,31)	-0,2	0,81 (0,54-1,20)	-1,04 (0,29)
Alcohol	-0,33	0,71 (0,49-0,75)	-2,47 (0,001)*	-0,05	0,94 (0,87-1,01)	-1,37 (0,11)

SES: nivel socioeconómico

* p < 0,05; sl: significancia limítrofe

## Asociación del consumo de alcohol con variantes genéticas

En el cuadro 6 se observan los resultados de dos variantes del gen *ALDH1A1 (rs8187876* y *rs4646543)* y del gen *FTO (rs9939609* y *rs11075997).* En el cuadro 7 se presenta un resumen de las características genéticas más relevantes de dichos marcadores en la muestra.

**Cuadro 6:** Efecto de las variables genéticas sobre el consumo de alcohol y análisis de interacciones con el índice de masa corporal

Marcador	Alelo de riesgo	Modelo aditivo cantidad	Modelo aditivo frecuencia	Modelo dominante cantidad	Modelo dominante frecuencia
		ß (p)	ß (p)	ß (p)	ß (p)
*rs8187876*	C Vs. A	0,35 (0,06)	0,44 (0,05)	-0,04 (0,92)	-0,04 (0,88)
*rs4646543*	T Vs. C	0,39 (0,01)*	0,2 (0,01)*	0,7 (0,002)*	0,33 (0,01)*
*rs11075997*	C Vs. T	-0,08 (0,91)	0,47 (0,46)	0,07 (0,60)	0,33 (0,75)
*rs9939609*	A Vs. T	0,01 (0,99)	0,01 (0,92)	-0,09 (0,84)	0,01 (0,92)
IMC x *rs8187876*		-0,01 (0,98)	-0,16 (0,91)	-0,01 (0,80)	2,33 (0,54)
IMC x *rs4646543*		0,24 (0,02)*	0,05 (0,11)	0,17 (0,31)	0,02 (0,76)
IMC x *rs11075997*		0,17 (0,12)	0,01 (0,74)	0,11 (0,57)	-0,01 (0,78)
IMC x *rs9939609*		0,05 (0,61)	0,02 (0,63)	0,13 (0,43)	0,03 (0,64)

* p < 0,05

**Cuadro 7:** Frecuencias alélicas y genotipos de los marcadores genéticos

	Alelos	Frecuencia alélica	Genotipo	Frecuencia genotípica		Prueba de Hardy- Weinberg	Distribución entre sexos	Distribución entre clases según IMC	
				Obs	Esp			Sobrepeso	Obesidad
*rs8187876*	A	0,21	AA	0,05	0,04	χ^2^ = 0,46, p = 0,79	χ^2^ = 3,41, p = 0,18	χ^2^ = 5,07, p = 0,08	χ^2^ = 4,83, p = 0,08
	C	0,79	AC	0,31	0,33				
			CC	0,64	0,63				
*rs4646543*	T	0,52	TT	0,28	0,27	χ^2^ = 0,05, p = 0,97	χ^2^ = 0,01, p = 0,99	χ^2^ = 2,70, p = 0,25	χ^2^ = 1,42, p = 0,49
	C	0,48	TC	0,49	0,50				
			CC	0,23	0,23				
*rs9939609*	T	0,63	TT	0,45	0,39	χ^2^ = 6,22, p = 0,045*	χ^2^ = 7,5, p = 0,02*	χ^2^ = 1,88, p = 0,38	χ^2^ = 10,01, p < 0,01*
	A	0,37	TA	0,37	0,47				
			AA	0,18	0,14				
*rs11075997*	T	0,51	TT	0,23	0,26	χ^2^ = 1,15, p = 0,56	χ^2^ = 0,20, p = 0,90	χ^2^ = 3,76, p = 0,15	χ^2^ = 3,63, p = 0,16
	C	0,49	TC	0,54	0,54				
			CC	0,23	0,23				

IMC: índice de masa corporal; Obs: observado; Esp: esperado

* p < 0,05

Se realizaron dos modelos respecto a la cantidad de alcohol consumido, uno aditivo y uno dominante. Se encontró que el marcador *rs4646543* fue significativo en los dos modelos, siendo T el alelo de riesgo. Para *rs8187876,* solamente el modelo aditivo fue significativo para la frecuencia de consumo, siendo C el alelo de riesgo, aunque el valor de significancia fue muy ajustado (p = 0,05). Los otros modelos fueron negativos para este marcador. Las variantes del gen *FTO* no presentaron diferencias significativas según el grado de alcohol de la muestra.

En el estudio de interacción, el efecto estadístico observado con el índice de masa corporal sobre el alcohol no se vio modificado por las variables estudiadas, como sexo, edad, nivel socioeconómico, actividad física, grado de educación o alguna de las variantes genéticas. Sin embargo, se encontró un efecto de interacción entre el IMC y los alelos de *rs4646543* cuando se consideraron solamente las personas bebedoras (p = 0,02), lo que podría indicar que el efecto del peso sobre la cantidad de alcohol es mayor en personas con el alelo de riesgo.

## Discusión

La relación entre el exceso de peso y el consumo de alcohol es un tema de sumo interés para la salud pública y ha sido objeto de estudio en las últimas décadas en varios países, con diversos tamaños de cohortes y en varias regiones etnogeográficas ^8^^,^^18^^-^^20^.

En Argentina se han realizado pocos estudios sobre el tema. Los resultados del presente trabajo indicaron que el consumo de alcohol en cantidades moderadas está inversamente asociado con el peso corporal, relación que resultó estadísticamente significativa en el sexo femenino.

De manera similar, en otro estudio realizado en la ciudad de Córdoba, se encontró que las personas que tomaban menos de 100 ml de bebidas alcohólicas al día tenían menos riesgo de sobrepeso u obesidad. El riesgo reportado fue similar al presentado aquí: OR = 0,7 ^21^. En otro estudio en la misma ciudad, cuyo objetivo primario era identificar patrones dietéticos a partir del análisis de componentes principales, se encontró que uno de los cuatro patrones identificados era la relación positiva entre aperitivos y bebidas alcohólicas, respaldando el papel estimulante que tiene el alcohol sobre la ingestión de comidas saladas ^22^.

Recientemente, Lara y Serio analizaron la asociación entre los valores de IMC y el consumo de alcohol a partir de las Encuestas Nacionales de Factores de Riesgo (ENFR), en el contexto de la prevalencia de hipertensión arterial sistémica. Las autoras encontraron una relación estrecha y positiva entre la cantidad de copas por día y el aumento del IMC, especialmente en personas con IMC mayor de 30 kg/m^2^ y en mujeres. Sin embargo, no disgregaron los efectos del consumo leve o moderado (menos de una o dos copas), por lo que se pudo enmascarar algún efecto protector en los datos de la encuesta ^23^.

En otro estudio recientemente publicado, se reportó que el consumo de alcohol está asociado con un incremento en el riesgo de sobrepeso en estudiantes de las universidades de Argentina, México y Paraguay, pero no en las de Chile, Guatemala y Panamá, donde el efecto fue protector. Cabe destacar que la información se obtuvo mediante formularios autocompletados por redes sociales ^24^. A pesar de estos resultados dispares, una característica común en la mayoría de los estudios es que la mayor prevalencia de consumo de alcohol se registra en los hombres, con excepción del estudio de Silva-Fonseca *et al.,* de 286 estudiantes de primer año de ciencias médicas de la Universidad Federal de Río de Janeiro, Brasil ^25^.

La relación inversa entre el valor de IMC y la frecuencia de tragos por semana puede estar afectada por la elección de la bebida y la cantidad de consumo ^26^. Los resultados deben interpretarse cuidadosamente, ya que se trata de un estudio transversal y no hay una relación temporal entre exposiciones y efectos. Una posible fuente de confusión es la edad, ya que el consumo de alcohol es más frecuente en mujeres jóvenes, quienes suelen tener menor peso corporal.

Esta objeción fue nombrada en otros análisis como posible fuente de error, pero no parece ser el caso del presente estudio, pues no se encontraron diferencias significativas de consumo entre grupos de edad mediante el análisis de Kruskal-Wallis y las regresiones lineales no fueron significativas para la variable edad. Además, en estudios longitudinales en los que el diseño minimiza el efecto de la edad, también se encontró dicha relación inversa ^27^. En este sentido, un estudio longitudinal de casi 50.000 mujeres sin antecedentes de enfermedades cardiovasculares, seguidas durante diez años, demostró que aquellas que tomaban hasta 4 g de alcohol al día, tenían 8 % menos riesgo de sobrepeso respecto a las abstemias; no obstante, por encima de 4 g diarios de alcohol, el riesgo de sobrepeso aumentaba hasta cinco veces ^28^.

Se han propuesto varios mecanismos por los cuales el alcohol influye en la ganancia de peso. Este puede estimular la secreción de hormonas del apetito, incrementar la ingestión calórica, promover la termogénesis y modificar el gasto energético ^29^^,^^30^. Un aspecto para resaltar es que las personas que consumen alcohol en cantidades moderadas suelen ser más conscientes de su salud y, en general, tienen un patrón de comportamiento más saludable ^19^. Sin embargo, al igual que en el presente estudio, se reporta que la relación inversa entre alcohol y peso se mantiene aun después de ajustarla por actividad física y otros factores ^31^.

Con respecto al vino, se sabe que algunos de sus componentes tienen la cualidad de inducir la expresión de aromatasas y otros intermediarios en el tejido adiposo ^32^. Por ejemplo, el resveratrol, un potente antioxidante natural, podría influir sobre el efecto de las hormonas, regulando el tamaño y el metabolismo de los adipocitos ^33^. Esto no explica el efecto potencial de la cerveza, que también tuvo una relación similar con el IMC. En un estudio en la República Checa, donde la cerveza es la bebida principal y no está asociada con un determinado nivel sociocultural como suele suceder en Estados Unidos o Francia, Bobak *et al.* encontraron una asociación negativa entre su consumo y la ganancia de peso cuando se estudiaron mujeres, y una positiva, cuando se consideraron los hombres ^34^.

En Argentina, la cerveza es una bebida tradicional y en los últimos años se ha vuelto tendencia su consumo y, más aún, el de las fabricadas regionalmente y en pequeñas cantidades. Aunque hay pocos estudios sobre los efectos de este tipo de cervezas en comparación con las de marcas comerciales, estos sostienen que la abundancia de compuestos polifenólicos es beneficiosa para la salud ^35^.

Cuando se analizaron los genes del metabolismo del alcohol, se determinó que el alelo T en la variante *rs4646543* del gen *ALDH1A1* está asociado con mayor frecuencia y cantidad de consumo de alcohol. La presencia del alelo T en la variante *rs4646543* estuvo asociada con un aumento de 0,39 unidades de alcohol respecto al alelo C, manteniéndose las demás variables constantes (ß = 0,39; p = 0,01), y además, con un aumento de la frecuencia semanal de 0,2 episodios (ß = 0,2; p = 0,001). Dada su función desintoxicante, las variantes de este gen poseen varios efectos biológicos, incluido el enrojecimiento al tomar alcohol, la sensibilidad a la bebida y la dependencia a las drogas ^36^.

En un trabajo presentado por Liu *et al.,* se reportó que los haplotipos de *ALDH1A1* difieren significativamente en su asociación con el alcohol en poblaciones de diferentes orígenes, como las nativo-americanas, caucásico-finlandesas y afroamericanas ^37^. También, se está indagando sobre la respuesta de los alelos de riesgo de *ALDH1A1* a diferentes fármacos; sus polimorfismos se asocian con una mayor mortalidad en mujeres hispanas con cáncer de mama ^38^. Una de las actividades más estudiadas de este gen es la regulación del tejido adiposo y su papel en la obesidad. La *ALDH1A1* es una enzima asociada directamente con la degradación de la dopamina en el cerebro, la que sucede en el área que gobierna el equilibrio de la motivación y la recompensa ^39^^,^^40^. En los adipocitos, la expresión de este gen está involucrada en el aumento de la apoptosis y la inhibición de la adipogénesis, ^41^, dos procesos clave que podrían resultar en termogénesis y en eventual reducción de la grasa ^42^^,^^43^.

En el presente trabajo, se encontraron indicios sobre una interacción potencial entre los genotipos de riesgo de *ALDH1A1* y el IMC, al analizar la cantidad de alcohol consumido. Sin embargo, dicha interacción debe interpretarse con cuidado porque estuvo limitada a las personas bebedoras y no se repitió en todos los modelos.

Es crucial reconocer las limitaciones de este estudio. En este caso, el tamaño de la muestra podría limitar la representatividad de las frecuencias alélicas y aumentar la posibilidad de error. Otras desventajas son el limitado número de marcadores y el diseño del estudio porque no permite hacer relaciones del tipo causa-efecto. A pesar de esto, los resultados obtenidos son relevantes y sirven como punto de partida para trabajos subsiguientes. En este sentido, los autores están planificando una nueva campaña, ampliando el tamaño muestral, el rango de consumo y el número de marcadores analizados.

En conclusión, el determinar la influencia que posee el alcohol sobre el sobrepeso es complejo por la naturaleza multifactorial de la condición y por la variabilidad inherente a cada población. Los datos del presente trabajo sugieren que las bebidas alcohólicas, tomadas moderadamente y en episodios espaciados, no contribuyen a un mayor peso corporal o, inclusive, tienen una relación inversa, particularmente en mujeres. Aunque no hay un consenso sobre los mecanismos que subyacen en este comportamiento, una posibilidad es que ciertas variantes de los genes desintoxicantes, como *ALDH1A1* , posean efectos adicionales sobre la regulación del metabolismo del tejido adiposo o el control del apetito.
